# Evaluation of carbonic anhydrase IX as a therapeutic target for inhibition of breast cancer invasion and metastasis using a series of *in vitro* breast cancer models

**DOI:** 10.18632/oncotarget.4498

**Published:** 2015-07-03

**Authors:** Carol Ward, James Meehan, Peter Mullen, Claudiu Supuran, J. Michael Dixon, Jeremy S. Thomas, Jean-Yves Winum, Philippe Lambin, Ludwig Dubois, Nanda-Kumar Pavathaneni, Edward J. Jarman, Lorna Renshaw, InHwa Um, Charlene Kay, David J. Harrison, Ian H. Kunkler, Simon P. Langdon

**Affiliations:** ^1^ Division of Pathology, Institute of Genetics and Molecular Medicine, University of Edinburgh, Edinburgh, United Kingdom; ^2^ School of Medicine, University of St Andrews, North Haugh, St Andrews, United Kingdom; ^3^ Università degli Studi di Firenze, Polo Scientifico, Laboratorio di Chimica Bioinorganica, Sesto Fiorentino, Florence, Italy; ^4^ Edinburgh Breast Unit, Western General Hospital, Edinburgh, United Kingdom; ^5^ Department of Pathology, Western General Hospital, Edinburgh, United Kingdom; ^6^ Institut des Biomolécules Max Mousseron (IBMM), UMR 5247, CNRS-UM1-UM2, Batiment de Recherche Max Mousseron, Ecole Nationale Supérieure de Chimie de Montpellier, Montpellier, France; ^7^ Department of Radiation Oncology (MaastRO), GROW-School for Oncology and Developmental Biology, Maastricht University Medical Center (MUMC+), Maastricht, The Netherlands; ^8^ Edinburgh Cancer Research Centre, Institute of Genetics and Molecular Medicine, University of Edinburgh, Edinburgh, United Kingdom

**Keywords:** carbonic anhydrase IX, breast cancer, tumour microenvironment, invasion, hypoxia

## Abstract

Triple negative, resistant or metastatic disease are major factors in breast cancer mortality, warranting novel approaches. Carbonic anhydrase IX (CAIX) is implicated in survival, migration and invasion of breast cancer cells and inhibition provides an innovative therapeutic strategy. The efficacy of 5 novel ureido-substituted sulfamate CAIX inhibitors were assessed in increasingly complex breast cancer models, including cell lines in normoxia and hypoxia, 3D spheroids and an *ex-vivo* explant model utilizing fresh biopsy tissue from different breast cancer subtypes. CAIX expression was evaluated in a tissue microarray (TMA) of 92 paired lymph node and primary breast cancers and 2 inhibitors were appraised *in vivo* using MDA-MB-231 xenografts. FC11409B, FC9398A, FC9403, FC9396A and S4 decreased cell proliferation and migration and inhibited 3D spheroid invasion. S4, FC9398A and FC9403A inhibited or prevented invasion into collagen. FC9403A significantly reversed established invasion whilst FC9398A and DTP348 reduced xenograft growth. TMA analysis showed increased CAIX expression in triple negative cancers. These data establish CAIX inhibition as a relevant therapeutic goal in breast cancer, targeting the migratory, invasive, and metastatic potential of this disease. The use of biopsy tissue suggests efficacy against breast cancer subtypes, and should provide a useful tool in drug testing against invasive cancers.

## INTRODUCTION

A recent analysis by over 100 breast cancer specialists emphasized critical gaps in research knowledge needed to increase the successful treatment of breast cancer. Targeting the tumour microenvironment, novel therapies for metastatic and triple negative breast cancer and better experimental models that encompass breast cancer subtypes were amongst the factors highlighted [1]. To address several of these issues, we examined the effect of novel inhibitors of carbonic anhydrase IX (CAIX), an enzyme involved in pH regulation, on breast cancer growth and invasion using a series of increasingly complex models.

As solid tumors develop, areas of low oxygen tension and acidosis are produced which are associated with the development of radiation and chemotherapy resistance [2]. Hypoxic conditions activate the transcription factor Hypoxia Inducible Factor-1 (HIF-1), a heterodimer containing HIF-1α and HIF-1β subunits [3]. HIF-1α expression is mainly dependent on hypoxia since, in the presence of oxygen, it is degraded within minutes [4]. However, dysregulation of tumor suppressors, oncogenes and growth factor receptor number increase the expression of HIF-1α and up-regulation of transcriptional targets in normoxic cancer cells [5, 6]. In breast cancer, increased HIF-1α expression is associated with poor prognosis, since HIF-1 controls expression of proteins required for surviva1 in hypoxic and acidic conditions [7, 8].

One such protein, CAIX, has been linked to poor prognosis and survival of breast cancer patients [9–12]. Expression of CAIX is low in normal breast tissue or benign lesions, but strongly expressed in 50% of all ductal carcinoma in situ (DCIS) of the breast, and in 38% of DCIS associated with invasive disease and is present mainly in abnormal epithelium in breast carcinoma [9, 13].

The CAIX transmembrane protein consists of a proteoglycan-like domain and an intracellular carboxy terminal tail which can influence cell-cell adhesion [14], and catalyzes the reversible reaction CO_2_ + H_2_O → H^+^ + HCO_3_^−^ [15]. This production of protons acidifies the tumor microenvironment, while bicarbonate ions are transported into the cell maintaining an alkaline intracellular pH [16, 17], which may inhibit apoptosis [2, 18].

Hypoxia increases expression of metalloproteinases in breast and other cancer cells; these enzymes are activated in acidic conditions causing destruction of basement membrane components thus enhancing the ability of tumor cells to metastasize [18, 19]. Similarly, cell migration depends on acidic conditions in lamellipodia, which are partially produced by activation of CAIX and its interaction with bicarbonate transporters [20]. Since the acidic/hypoxic conditions in the tumor microenvironment facilitate tumor progression, CAIX plays an important part in the acidification of the tumor environment and tumor metastasis. Metastatic growth is associated with epithelial to mesenchymal transition (EMT) and is strongly linked to loss of E-cadherin [21] because hypoxia activates the lysyl oxidase-Snail pathway which represses E-cadherin expression [22]. Furthermore, CAIX is known to impede E-cadherin mediated cell adhesion by decreasing the amount of E-cadherin bound to the cytoskeleton [14]. Recent studies also demonstrate that CAIX enhances the metastatic potential of tumor cells by a mechanism associated with decreased Rho-GTPase activity leading to EMT [23].

We and others have been involved in the development of novel, more specific CAIX inhibitors as a potential, innovative therapeutic strategy for treatment of breast cancer and other solid tumors [24–27]. However, targeting survival mechanisms involved in adaptation to the tumor microenvironment in oncology drug development requires culture techniques that reproduce these acidic/hypoxic conditions, and therefore more appropriate systems are needed to develop these approaches. We utilized a range of pre-clinical models to assess the effects of these compounds, from cell lines cultured in normoxic or hypoxic conditions to 3D spheroid culture and xenografts. This approach was extended by examining the effects of these compounds on the survival and invasion of *ex vivo* cultures of tumor explants from fresh, pre-treatment, breast cancer patient biopsies, which more accurately reflect receptor and oncogene overexpression and contain stromal elements that may affect therapeutic outcomes. This model system encompasses the heterogeneity found in breast tumors *per se*, as well as that found between different breast cancer subtypes.

Our data using these varied *in vitro* and *in vivo* experimental systems show that CAIX inhibition has a significant effect on migration, invasion and proliferation in the common breast cancer subtypes and that CAIX expression significantly correlates with metastasis in a series of lymph node positive patient breast tumors. This suggests that CAIX inhibitors are likely to form a useful therapeutic adjunct to conventional adjuvant radiotherapy and systemic therapy for breast cancer.

## RESULTS

### Novel ureido-sulfamate CAIX inhibitors reduce cell proliferation in a panel of breast cancer cell lines in normoxic and hypoxic conditions

**Figure 1:**
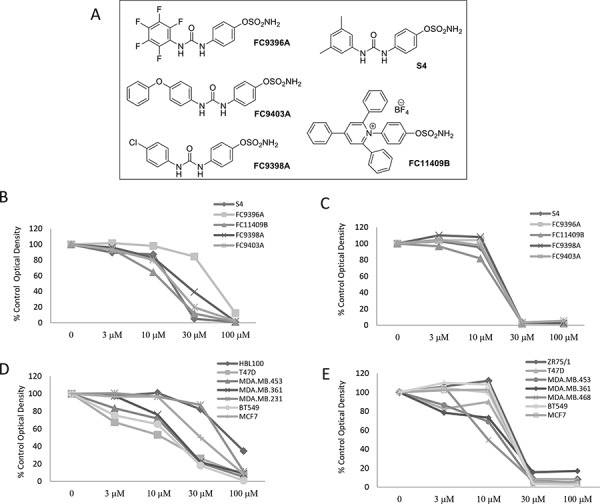
The anti-proliferative effects of novel ureido-sulfamate CAIX inhibitors on the growth of breast cancer cell lines *in vitro* **A.** The structures of the 5 novel ureido-sulfamate CAIX inhibitors, FC9396A, FC9403A, FC9398A, FC11409B, and S4 used in this study. **B.** The effect of the CAIX inhibitors, FC9396A, FC9403A, FC9398A, FC11409B, and S4 on the proliferation of MDA-MD-231 breast cancer cells in normoxia. Compounds were used at concentrations between 3 – 100 μM. Data represents mean of *n* = 5 assays; 6 replicates per assay, at day 5. **C.** The response to the CAIX inhibitors, FC9396A, FC9403A, FC9398A, FC11409B, and S4 on the proliferation of MDA-MD-231 breast cancer cells in hypoxia (0.5% O_2_) Compounds were used at concentrations between 3 – 100 μM. *n* = 2 assays, 6 replicates per assay at day 5. **D.** The concentration response of a panel of various breast cancer cell lines to the CAIX inhibitor FC9396A in normoxic conditions. Compounds were used at concentrations between 3 – 100 μM. *n* = 3 assays, 6 replicates per assay at day 5. **E.** The anti-proliferative response of a panel of breast cancer cell lines to the CAIX inhibitor FC9398A used at concentrations between 3 – 100 μM, in hypoxia (0.5% O_2_). *n* = 2 assays, 6 replicates per assay at day 5.

Our previous study evaluating a series of 50 novel ureido-sulfamate CAIX inhibitors, showed that 5 compounds could significantly inhibit proliferation of several human breast cancer cell lines in both hypoxic (0.5% O_2_) and normoxic conditions (21% O_2_); these were FC11409B, FC9398A, FC9403, FC9396A and S4 (24). Their structures are shown in Figure 1A. K_i_ values for these compounds against CAIX and details of synthesis were previously reported [25]. We examined the effects of these inhibitors in an expanded panel of breast cancer cell lines (Supplementary Table S1) representing the major breast cancer subtypes, with variable hormone and growth factor receptor expression. The effect of all 5 compounds on cell proliferation is illustrated using the MDA-MB-231 cell line as an example in Figure 1B (normoxia) and Figure 1C (hypoxia). Similar data were obtained for all cell lines examined. In normoxic conditions the inhibitory effect of all 5 compounds on cell proliferation in this cell line was highly significant (*P* < 0.001) at 30 and 100 μM except for FC9396A which was significant at 100 μM alone and for FC9403 which significantly inhibited cell proliferation at 10 μM (Figure 1B). The variation in response between some of the cell line panel in normoxia is demonstrated in Figure 1D using FC9396A as an example. The dose responses demonstrated that the IC_50_ values for each compound varied between cell lines in normoxic conditions (Supplementary Table S2). However, in hypoxic conditions, the effect of all the inhibitors was highly significant (*P* < 0.001) at 30 and 100 μM, with cell proliferation almost completely inhibited at these concentrations (Figure 1C). The responses to all compounds in low oxygen tensions were similar in each of the cell lines with IC_50_ values between 10 and 30 μM. For illustration, the variation of response to one inhibitor FC9398A under hypoxic conditions in the cell line panel is shown in Figure 1E.

### The effect of novel CAIX inhibitors on breast cancer cell migration in hypoxia and normoxia

The influence of the five CAIX inhibitors on migration was assessed using wound healing assays with the MDA-MB-231 invasive cell line. The results for S4 in normoxia (Supplementary Figure S1A) and hypoxia (Supplementary Figure S1B) show concentrations of 30 μM clearly inhibit migration. Supplementary Table S3 illustrates that these novel CAIX inhibitors reduce the migratory capacity of these cells under both normoxic and hypoxic conditions, except for FC9396A, which was only effective in normoxia. However, MDA-MB-231 cells migrated into the wound area as single cells, making accurate quantitation difficult. Therefore the same inhibitors were further assessed using the invasive ovarian cell line SKOV3, to determine firstly, that the effects on migration/invasion are not specific to the MDA-MB-231 model alone and also to provide quantitative data (Supplementary Figure S1C).

Significant inhibition of motility was observed in SKOV-3 cells treated with S4 at 10, 30, and 100 μM in normoxic conditions and at 30 and 100 μM in hypoxic conditions. FC11409B inhibited migration in both normoxia and hypoxia, but only at the highest concentrations of 100 μM. FC9396A had little inhibitory activity in this assay in normoxic or hypoxic conditions, while FC9398A, was effective at 100 μM in normoxia, and 30 and 100 μM in hypoxia. FC9403A was equally effective regardless of oxygen status at both 30 and 100 μM. The combined results for this assay suggest that the compounds S4 and FC9403A are the most active inhibitors of migration.

### CAIX expression in breast cancer cell 3D spheroids

3D breast cancer spheroids, which contain a graduated range of oxygen concentrations, were assessed to determine the presence of hypoxic areas and CAIX positive staining. The hypoxic core of an MDA-MB-231 spheroid is shown using ‘Hypoxyprobe’ visualized using a fluorescent microscope (Supplementary Figure S2A). Similarly, HIF-1α staining is also shown for comparison in MDA-MD-231 spheroids (Supplementary Figure S2B). Immunohistochemical staining for CAIX in several breast cancer cell spheroid models showed that the expression of this enzyme is not confined to hypoxic areas (Supplementary Figure S2C). Although it has previously been reported that CAIX expression is confined to the hypoxic cores of 3D spheroids [17], we clearly show that in many of our breast cancer cell spheroid models, that there is variant expression of CAIX in areas of the spheroids that are not hypoxic (Supplementary Figure S2C).

### The effect of CAIX inhibitors on 3D breast spheroid invasion

**Figure 2:**
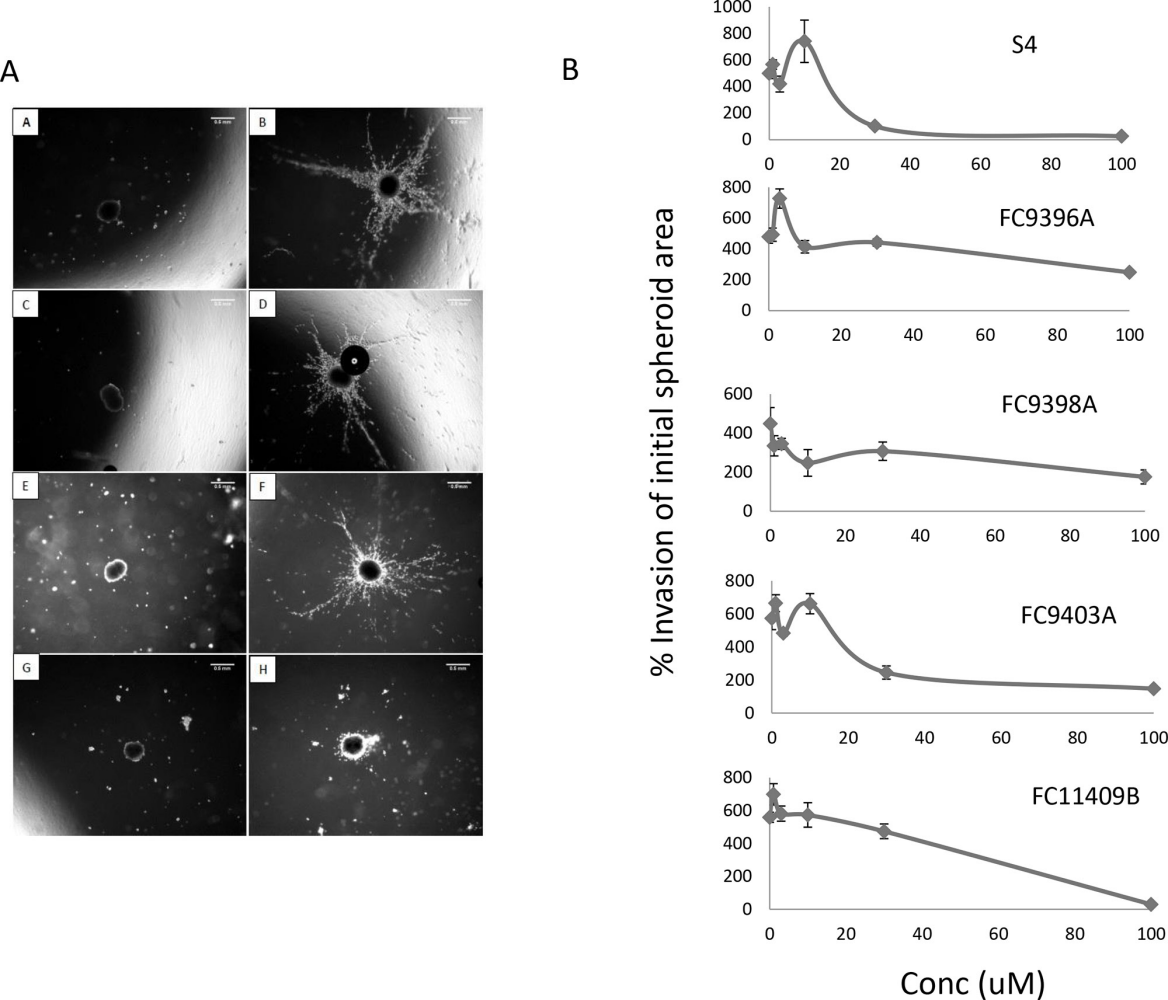
The effect of S4, FC9396A, FC9403A, and FC11409B on the ability of MDA-MB-231 spheroids to invade collagen **A.** 3D collagen invasion assay. Shown are the results of treating MDA-MB-231 spheroids with the CAIX inhibitor FC11409B at time 0 and at 48 h. A, C, E and G are time 0; B, D, F, H, are time 48 hours. A and B are controls; C and D, FC11409B 10 μM; E and F, FC11409B 30 μM; G and H, FC11409B 100 μM. Original magnification of images = × 25. Scale bar = 0.5 mm. **B.** The effects of S4, FC9396A, FC9403A, and FC11409B on the ability of MDA-MB-231 spheroids to invade collagen. Inhibitors were used at 0, 3, 10, 30 and 100 μM. The invasive areas of each of the spheroids treated with the various concentrations of drug were normalized against the invasion area of the control spheroids. Results shown are mean ± SEM, *P < 0.01. *n* = 8 for all drug concentrations.

To examine the ability of the CAIX inhibitors to impede invasion of 3D breast cancer cell cultures, MDA-MB-231 spheroids were embedded in collagen plugs containing the CAIX inhibitors at different concentrations and examined at 24 and 48 h. A representative experiment is shown in Figure 2A (A–H), and results for all compounds are shown in Figure 2B. This data reveals significant inhibition in the capacity of MDA-MB-231 spheroids to invade a collagen matrix if treated with S4 or FC9403A at 30 or 100 μM and FC11409B, FC9398A, FC9396A at 100 μM. FC9396A (3 μM) however, significantly increased invasion. Although both 2D and 3D migration/invasion assays showed some inhibitory activity for all the active compounds, the results from 3D spheroid collagen invasion assays confirmed that S4 and FC9403A are the most active inhibitors of migration/invasion.

### The effect of CAIX inhibitors on growth and invasion of tumor explants

**Figure 3:**
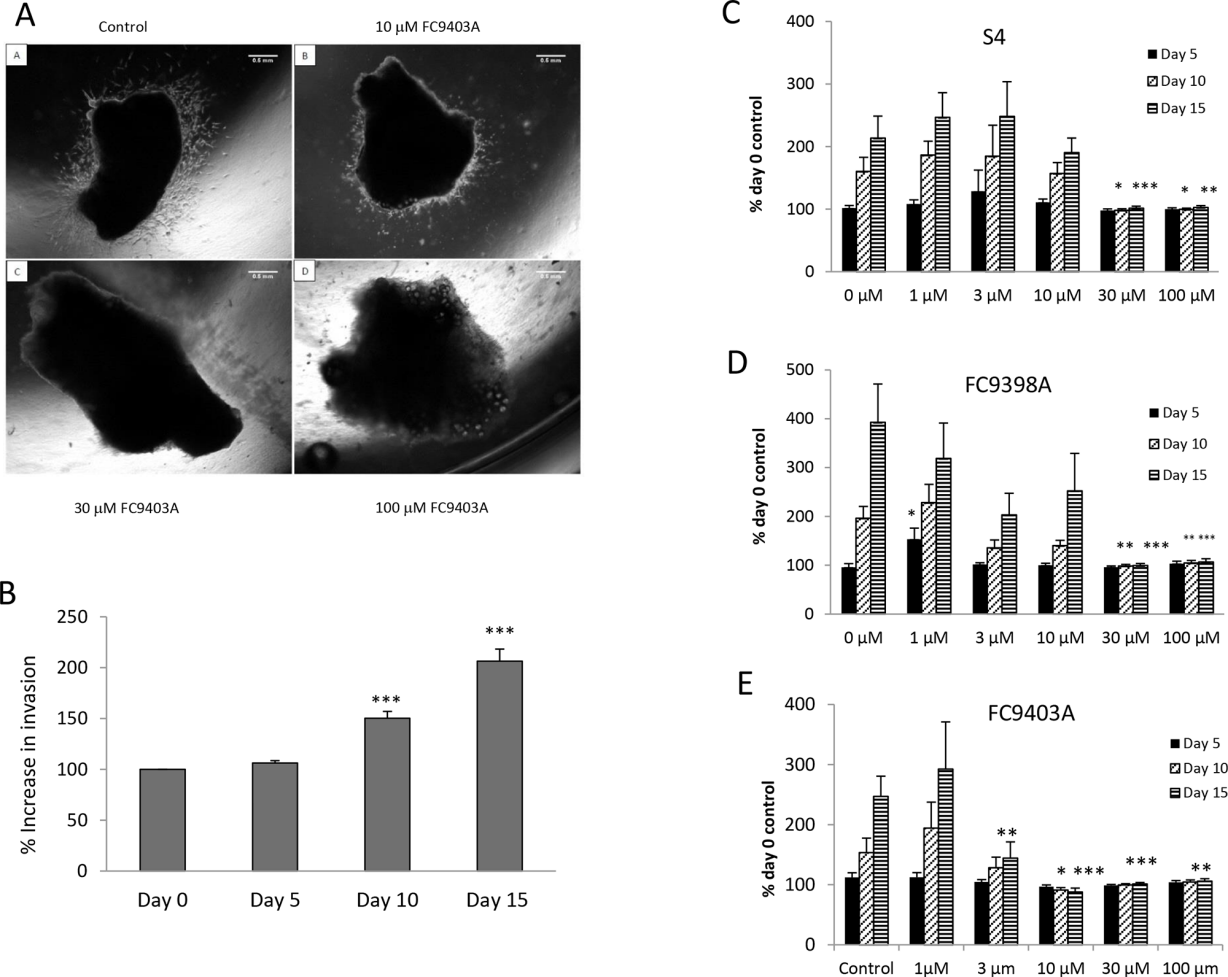
The effects of S4, FC9398A and FC9403A on breast cancer invasion within an explant invasion assay **A.** Breast tumor explant invasion assays. Representative experiment showing the ability of FC9403A to inhibit the invasion of an ER+ breast tumor explant into collagen after 5 days. Magnification × 25. Scale bar = 0.5 mm. **B.** Collagen embedded explants showing control invasion over 15 days when measured by increased area. Data pooled from 26 separate biopsy samples (*n*= 214 – 177). Results shown = mean ± SEM, ***P < 0.001. **C–E.** Inhibition of invasion of collagen-embedded explants. C) S4 treatment; D) FC9398A treatment; E) FC9403A treatment. Explants were cultured for 15 days with measurements taken every 5 days. Mean ± SEM (*n* = 8) 21-25 explants per condition *P < 0.05, **P < 0.01, ***P < 0.001 of relevant control

To assess the effects of CAIX inhibition in actual tumor tissue, *ex vivo* human breast tumor explants from pre-treatment fresh core needle biopsies were examined. A representative assay using FC9403A is shown in Figure 3A (A–D), after 5 days of culture, and demonstrates clear invasion in both the control (A) and 10 μM (B) treated explants, while higher concentrations of 30 (C) and 100 μM (D) prevented invasion. Figure 3B illustrates pooled data from 26 separate biopsy specimens (explant number = 214 – 177 per time point) after growth in collagen for 15 days with invasion assessed by increased area. Although approximately 50% of explants were invasive by Day 5, the percentage increase in invasion was significantly increased by Day 10. The effect of three CAIX inhibitors, S4, FC9403A and FC9398A, on growth and invasion were assessed.

The results for S4 (Figure 3C) show at day 5 there is no significant growth under any treatment. However by day 10 and 15 the higher concentrations of S4 (30 and 100 μM) have significantly prevented growth and invasion of the tumor tissue. Similar results were obtained using the compound FC9398A and FC9403A (Figure 3D and 3E). Again, by day 15 concentrations of FC9403A from 3 to 100 μM significantly inhibited explant invasion into collagen in comparison with untreated control explants.

**Figure 4:**
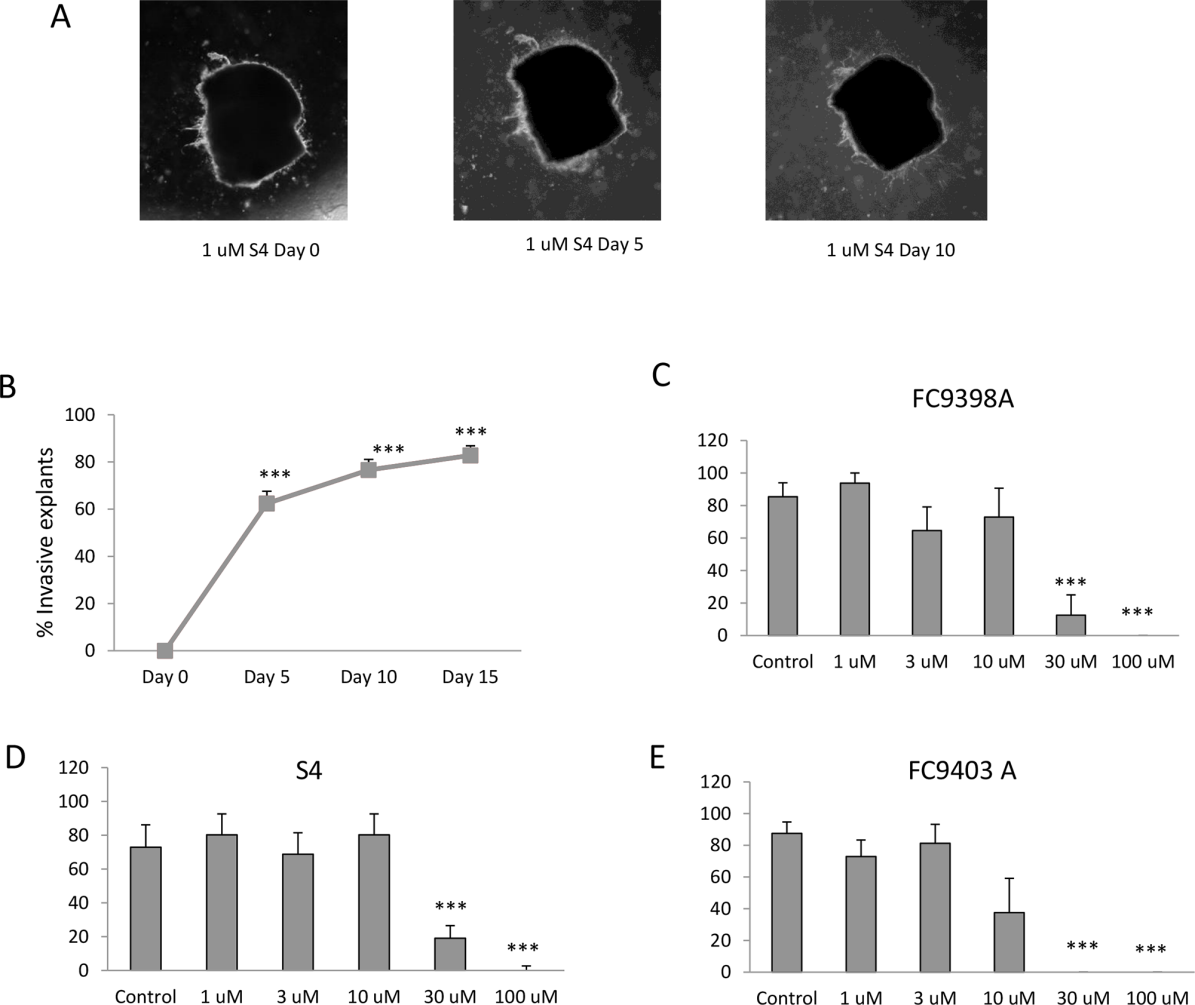
The effects of S4, FC9398A and FC9403A, and FC11409B on breast cancer invasion **A.** An example of decrease in tumor explant area in an invasive explant. Explant treated with 1 μM S4 over 10 days. Magnification × 25. **B.** Percentage of control explants showing invasive morphology over 15 days. Data shown are mean ± SEM (*n* = 230 explants). ***P < 0.001. **C.** Percentage of FC9398A treated explants showing invasive morphology. Explants cultured for 15 days with measurements taken every 5 days. Inhibitor was used at concentrations between 3 – 100 μM. Data shown are mean ± SEM *n* = 4. ***P < 0.001. **D.** Percentage of S4 treated explants showing invasive morphology. Explants cultured for 15 days with measurements taken every 5 days. Inhibitor was used at concentrations between 3 – 100 μM. Data shown are mean ± SEM *n* = 4. ***P < 0.001. **E.** Percentage of FC9403A treated explants showing invasive morphology. Explants cultured for 15 days with measurements taken every 5 days. Inhibitor was used at concentrations between 3 – 100 μM. Data shown are mean ± SEM *n* = 4. ***P < 0.001

Although some explants showed invasive capacity, they also demonstrated regression in actual explant size (Figure 4A). These would be considered non-invasive if explant area measurement was the sole criteria used to determine the effects of the CAIX inhibitors on invasion. Therefore, to ensure that invasion was measured accurately, we recorded whether or not each explant invaded collagen when treated with differing concentrations of each of the inhibitors (Figure 4B–4E). These results depict only whether an explant was scored as invasive or non-invasive, regardless of area. Using these criteria, all three drugs significantly inhibited invasion at 30 and 100 μM, however, invasion was completely prevented by FC9403A at these concentrations.

### The effect of CAIX inhibitors on reversal of explant invasion

**Figure 5:**
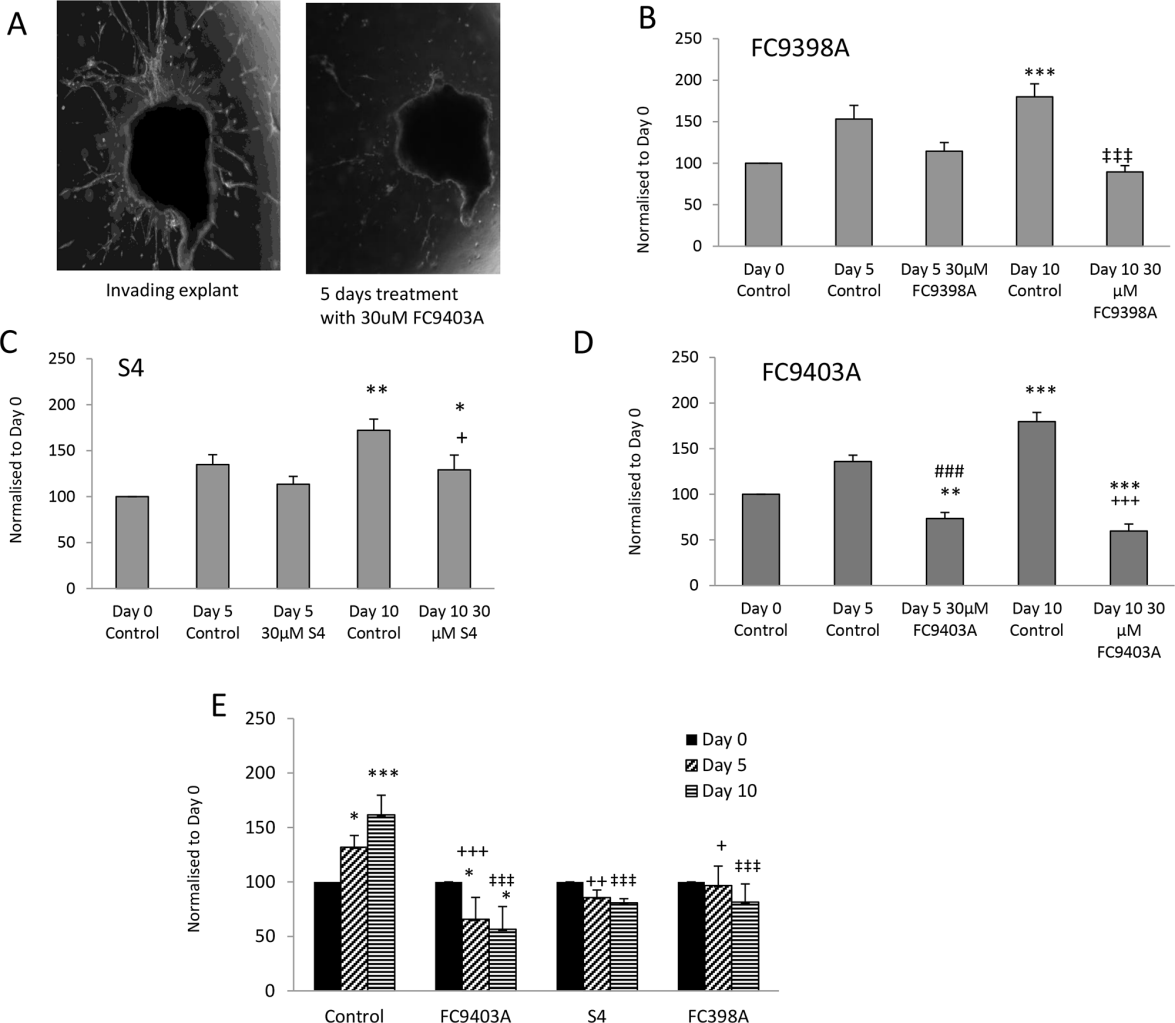
Reversal of invasion in human breast cancer explants after treatment using ureido-sulfamate CAIX inhibitors **A.** Representative images showing the reversal of invasion in a human breast cancer explant after 5 days of treatment using 30 μM FC9403A. Explants were monitored for 5 or 10 days and changes quantified using Image J. Original magnification × 25. **B.** The effect of FC9398A on explant invasion. Explants were allowed to invade into collagen plugs for 15 days, before treatment with 30 μM FC9398A. Data shown are mean ± SEM. *N* = 10 *P < 0.05; ***P < 0.001 compared to Day 0 control; ‡‡‡*P* < 0.001 compared to Day 10 control. **C.** The effect of S4 on explant invasion. Explants were allowed to invade into collagen plugs for 15 days, before treatment with 30 μM S4. Data shown are mean ± SEM. *N* = 10 − 13 *P < 0.05; **P < 0.05 compared to Day 0 control; + *P* < 0.05 compared to Day 10 control. **D.** The effect of FC9403A on explant invasion. Explants were allowed to invade into collagen plugs for 15 days, before treatment with 30 μM FC9403A. Data shown are mean ± SEM. *N* = 10 − 13 **P < 0.05; ***P < 0.001 compared to Day 0 control. ###*P* < 0.001 compared to Day 5 control; +++*P* < 0.001 compared with Day 10 control. **E.** Comparison of the effects of S4, FC9398A and FC9403A on invasive growth using the same biopsy tissue. Explants were allowed to invade into collagen plugs for 15 days, before treatment with 30 μM inhibitor. Data shown are mean ± SD. Samples in triplicate *P < 0.05; ***P < 0.001, compared to Day 0 control; ++*P* < 0.01; +++*P* < 0.001 compared with Day 5 control; ‡‡‡*P* < 0.001 compared with Day 10 control.

To determine whether these compounds could also influence established invasive growth, inhibitors were added after 15 days to invasive explants and the consequences monitored. A representative experiment using FC9403A is shown in Figure 5A. Results for FC9398A (Figure 5B) indicate that, although invasion has not been reversed, the inhibitor has prevented any further growth in the invasive front since there is no significant difference in invasive area after treatment with drug for 5 or 10 days when compared to the Day 0 (start of treatment) control. After 10 days treatment, there was a significant inhibition of growth in comparison with Day 10 control. Similarly, S4 did not reverse invasion over this timeline, but after 5 days treatment, there was no significant increase in growth in control or treated invasive explants (Figure 5C). However by day 10 growth of explants treated with 30 μM S4 was significantly different from both day 0 and day 10 controls, suggesting that although invasive growth was continuing, it was at a reduced rate. Interestingly, data for FC9403A indicated that growth was not only inhibited, but the invasive process was significantly reversed as illustrated in Figure 5A. Statistical analysis shows significant decreases in the invasive area of these explants after 5 and 10 days treatment with FC9403A (Figure 5D).

Because of the heterogeneous nature of the human biopsy tissue within the studies shown in Figure 5A–5D, and since separate biopsy material had been used for each inhibitor, a further experiment was performed using explant tissue from the same biopsy and the inhibitors compared using 30 μM of each inhibitor within the same treatment schedule (Figure 5E). This demonstrates that only explants treated with FC9403A showed a significant decrease in invasive area in comparison with Day 0 controls denoting a reversal of the invasive front, while both S4 and FC9308A have significantly prevented any further invasive growth at both time-points when inhibitor-treated explants are compared with Day 10 and 15 controls. These results are in accordance those shown in Figure 5A–5D.

### CAIX expression in a node positive breast cancer series

To confirm the relevance of these *in vitro* and *in vivo* findings, CAIX expression was assessed in a series of 92 breast cancer patients with involved lymph nodes from which a tissue microarray had been assembled. Significantly higher CAIX expression was found in triple negative breast cancer subtypes when compared to luminal ER^+^ breast cancers (Supplementary Figure S3), in accordance with other studies [11, 12, 28].

### CAIX inhibitors limit tumor explant growth by decreasing cell proliferation and increasing apoptotic cell death

Histological and immunohistochemical examination of tumor explants (*n* = 4) show large numbers of invaded cells in the collagen gels (Supplementary Figure 4A and 4B) and that S4 (30 and 100 μM) (Supplementary Figure 4C and 4D) inhibited invasion. Higher cellular CAIX staining was associated with invasion (Supplementary Figure 4E and 4F) and the majority of invading cells in these explants were clearly CAIX positive.

Treatment with S4 decreased the number of CAIX-positive cells, with staining greatly reduced in explants treated with 100 μM S4 compared to controls (compare Supplementary Figure S4G and S4H with Supplementary Figure S4E and S4F). This suggests that CAIX inhibitors may induce cell death in CAIX-positive cells, since S4 treatment also increased levels of cleaved caspase 3 expression in comparison to untreated control (Supplementary Figure S5A–S5F). Tumor regression or stasis may be generated via reduced proliferation. Controls revealed higher expression of the proliferation marker Ki67 in comparison to S4 treated explants, (Supplementary Figure S5G and S5H).

### CAIX inhibitors slow tumor growth *in vivo* by decreasing cell proliferation and increasing cell death

**Figure 6:**
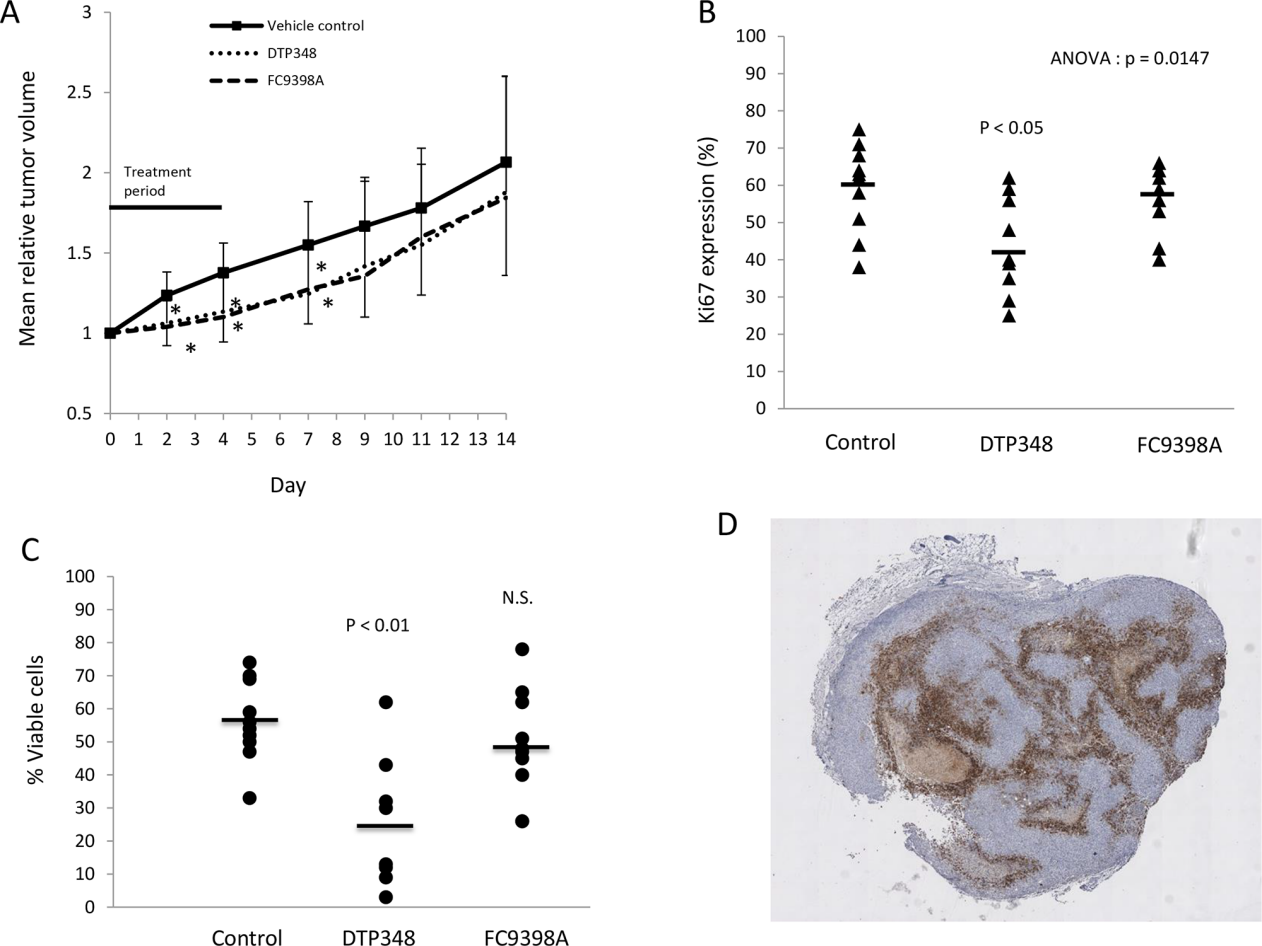
The effects of DTP348 and FC9398A on the MDA-MB-231 breast cancer xenograft model **A.** The effect of DTP348 and FC9398A on relative mean tumor volume of MDA-MB-231 breast cancer xenografts. 10 mice were used per treatment and tumors measured over a 14 day period. Data shown are mean ± SD. *p < 0.05 compared with day matched control. **B.** Ki67 expression in xenografts collected on final day (Day 14) of study. Mean values of approximately 2000 cells counted from multiple fields. **C.** Percentage viability in treated xenografts was assessed by use of Image J software on hematoxylin stained sections. **D.** CAIX staining in an untreated MDA-MB-231 xenograft. Magnification X25. Brown cellular areas indicate CAIX expression. Light brown acellular areas are areas of necrosis. Cells were stained with hematoxylin (blue).

Pharmacokinetic data (unpublished data-personal communication Erik Pettersen) indicated that FC9398A and a novel nitroimidazole based sulfamide CAIX inhibitor, DTP348 had better *in vivo* stability, with positive results in HT-29 colorectal xenograft models [26, 27]; (DTP348 structure is shown in [27]). MDA-MD-231 xenografts were therefore used to examine the *in vivo* effects of CAIX inhibition in breast cancer. Figure 6A illustrates that both these compounds could reduce relative tumor volume when used alone. Interestingly, although these two compounds had similar effects on tumor volume, only DTP348 significantly decreased Ki67 expression and reduced the percentage of viable cells in the xenograft model (Figures 6B and 6C). Figure 6D illustrates the extent and location of CAIX-positive cells in an untreated MDA-MB-231 xenograft.

## DISCUSSION

This study validates the pH regulator CAIX as a strong therapeutic candidate with potential effectiveness in treatment of breast cancer, including TNBC, and as far as we are aware, is the first report to show the preclinical efficacy of CAIX inhibition in human breast cancer tissue from various breast cancer subtypes, thus potentially helping to fill critical gaps in breast cancer treatment as previously outlined [1]. The effectiveness of several, novel, specific inhibitors was determined in a number of biological assays using both 2D and 3D cell and tissue models. We clearly demonstrate that these compounds can significantly inhibit and prevent breast cancer cell proliferation and invasion in a dose-dependent manner. Furthermore, the compound, FC9403A, could significantly reverse established invasion of *ex-vivo* collagen embedded fresh breast cancer biopsy material. The mechanisms involved in these effects depend on loss of proliferation and increased apoptosis of CAIX-positive cells.

Presently, there is a compelling requirement for better methods to control and treat metastatic disease, particularly in breast, lung and testicular cancers, which have greater metastatic potential than other malignancies [1, 29, 30]. In a murine knock down model, CAIX deficiency had no effect on growth, reproduction, health or lifespan of mice in comparison with wild type controls, and no morphologic or histologic effects other than gastric hyperplasia in targeted mice were observed [31, 32]. This suggests that inhibition of CAIX function should have limited toxicity in normal tissue supporting its potential as an anticancer therapy.

2D cultures demonstrated significant inhibition of cell proliferation in both normoxic and hypoxic conditions. In agreement with these results, an earlier study using RNAi, to decrease CAIX expression, found significant effects on growth and survival under both normoxic and hypoxic conditions in breast cancer cells [33]. Because oncogenic changes can stabilize HIF expression in normoxic cancer cells, [5, 6], CAIX expression can be increased in normoxic conditions in breast cancer cell lines (Supplementary Figure S2). A previous report has suggested that ureido-sulfamate inhibitors may preferentially target active CAIX [34], which may explain the more potent effect of these inhibitors in hypoxia, where CAIX activity would increase as cells become more reliant on glycolysis. Although others have described CAIX as being present only in the hypoxic cores of 3D spheroids [17], CAIX is clearly expressed independently of hypoxia and is not confined to the hypoxic peri-necrotic areas of breast cancer cell spheroids (Supplementary Figure S2). This may explain the effectiveness of these compounds in normoxic conditions in 2D assays, since some expression is clearly independent of oxygen concentration.

Wound healing assays illustrated the potential of 4 of these novel compounds, S4, FC11409B, FC9398A and FC9403A to effectively hinder migration of breast and ovarian cancer cells in both normoxic and hypoxic conditions (Supplementary Figure S1A–S1D). The C-terminal intracellular tail of CAIX is regulated by phosphorylation of Thr443 by PKA; this transmits signals to the extracellular enzyme domain in hypoxic conditions and influences cell migration [35, 36]. CAIX also increases cell migration and invasion *in vitro* via transcriptional activation and modulation of proteins involved in cytoskeletal reorganisation and EMT [23]. It has been postulated that CAIX interacts with the secreted protein DKK1 and interferes with the Rho/ROCK signaling pathway causing activation of paxillin, which promotes dynamic adhesion turnover and migration [23]. Interestingly, this Rho/ROCK pathway is pH sensitive [37, 38].

Spheroid invasion assays illustrated the potential of these CAIX inhibitors to hinder invasion and further, at higher concentrations, prevent it. Interestingly, the most invasive spheroids are those formed from MDA-MD-231 cells and as shown in Supplementary Figure S2C, these have extremely high CAIX expression. Using tumor explants from treatment naïve breast cancer patients, we confirmed that the inhibitors S4, FC9398A and FC9403A could inhibit and prevent metastatic invasion of collagen plugs using several methods of analysis (Figures 3 and 4). In addition, all these compounds could prevent further invasive growth of established explants and in the case of FC9403A, could significantly reverse invasive growth (Figure 5D and 5E). The majority of invasive cells from the explants were positive for CAIX expression. Treatment with inhibitory compounds increased the expression of cleaved caspase-3, while concomitantly reducing the expression of Ki67 and CAIX. Decreased expression of CAIX in treated explants implies that CAIX positive cells have been selectively removed, suggesting that apoptosis had been induced in cells expressing CAIX and that proliferation decreased in treated explants. Because FC9403A not only effectively inhibited tumor growth and invasion, but also reversed established invasive growth, the method involved in preventing invasion is not dependent on inhibition of metalloproteinases; supporting the involvement of apoptosis in removal of the invading cells.

Analysis of CAIX in a series of breast cancer patients with axillary node metastases showed that higher CAIX expression correlated with HER2 positive and triple negative breast cancer, which are prone to be more aggressive and metastatic. Several studies support a role for CAIX in breast cancer invasion and lymph node metastasis [11, 12, 39]. For example, IL-6/Notch-3 signaling increases CAIX expression and stimulates breast cancer cell invasion [40, 41] and HER2+ and triple negative breast cancers which express CAIX are associated with poor prognosis and high levels of early metastases to bone, lung and brain [42].

*In vivo* xenograft work, additionally demonstrated that these novel CAIX inhibitors can slow tumor growth by a mechanism involving decreased proliferation and loss of viability. This is supported by studies in a colon cancer model, where overexpression of CAIX amplified the growth rate of 3D spheroids and xenografts, but also increased necrosis, apoptosis and proliferation [43]. Increased growth caused greater areas of hypoxia in the core of the 3D structures, which would account for higher levels of cell death. This study also demonstrated that the area of viable tissue increased with CAIX expression [43]. CAIX knockdown has been shown to increase tumor regression, survival and relapse in a murine metastatic breast cancer model. Furthermore this strategy virtually eliminated metastatic growth [39]. The CAIX inhibitor S4, has also shown anti-metastatic capabilities in a murine model using the MDA-MB-231 breast cancer cell line [25].

Our data supports the premise that CAIX plays an essential role in the invasive and metastatic processes involved in breast cancer progression using several model systems. Importantly, this study shows in *ex vivo* assays using live human breast cancer tissue, that CAIX is a relevant therapeutic target for breast cancer treatment. As far as we are aware, this is the first report of this type, using live cultured *ex vivo* breast cancer tissue for a period of 25 days Recent work demonstrated that tumor grafts from breast cancer patients cultured in mice, accurately replicate tumor growth, metastatic potential and pathology of the major breast cancer classifications [44], and importantly, conserve the histological markers and features of the original tumor such as HER2 and ER positivity, as well as proliferation indices [45].

The explant *ex vivo* model reported herein, conserves many aspects of the original tumor pathophysiology and is therefore is more relevant for oncology drug trials, particularly those involving microenvironmental targets and further allows the influences of stromal elements on drug responses to be assessed. It not only reflects the heterogeneous nature of breast tumors *per se*, but also the heterogeneous nature of the disease, since biopsies from all major breast cancer subtypes were available for use in this study, suggesting that these CAIX inhibitors could be beneficial in all types of breast cancer.

Our results using these CAIX inhibitory compounds are supported by other recent findings reporting decreases in both primary tumor growth and metastasis in several preclinical breast tumor models with no signs of non-specific toxicity [25, 40, 46]. One CAIX inhibitor, Indisulam, is currently in Phase II clinical trials treating various tumor types, including stage IV melanoma, renal clear cell carcinoma, lung, pancreatic and metastatic breast cancer [47]. Our data clearly show that inhibition of CAIX using these novel ureido-sulfamate compounds influences both the proliferation/cell death and migration and invasion of human breast cancer cells. CAIX is therefore an attractive target for breast cancer treatment.

## MATERIALS AND METHODS

### Cell lines and culture

The cell lines used in this study, their receptor expression and breast cancer subtype are shown in Supplementary Table S1. Human breast cancer lines were cultured in DMEM supplemented with 10% fetal calf serum and 1% penicillin/streptomycin in normoxic (21%) or hypoxic (0.5%) O_2_ concentrations in a Don Whitley Hypoxystation H35 Workstation. MCF 10A cells were cultured in DMEM/F12, supplemented with donor horse serum (5%), epidermal growth factor (20 ng/ml) hydrocortisone (0.5 μg/ml), cholera toxin (100 ng/ml) insulin (10 μg/ml) and 1% penicillin/streptomycin. All cell lines were purchased from ATTC and tested and authenticated using STR profiling by Public Health England, Porton Down, Salisbury, UK, (November 2014).

### Sulforhodamine B assay

Cells were plated (1 × 10^3^/200 μl media) in 96-well plates and treated after 48 hours. At appropriate times, wells were fixed with 50 μl 25% trichloroacetic acid, washed with H_2_O and dried before addition of SRB (50 μl). After 30 minutes, plates were washed with 1% acetic acid. When dry, 150 μl of SRB assay buffer (10 mM Tris pH10.5), was added per well for 1 hour before reading at 540 nm on a Biohit BP800 platereader.

### Wounding/invasion assay

Cells (MDA-MB-231 or SKOV3 (5 × 10^5^/well)) were grown in 6-well plates until confluent. After wounding with a sterile tip, media containing 0.2% serum and the drug of interest were added. Invasion/migration of cells was calculated by comparison of the scratch at time ‘0′ and after 24 or 48 hours. The extent of migration of the SKOV3 cells was determined by analyzing the percentage of closed wound area, measured using DatInf Measure setup Wizard software.

### 3D cell culture

Cells were incubated in spinner flasks and cultured at 37°C in 5% CO_2_ until spheroids formed. Growth of live cells in 3D culture was monitored by adding a 10 μM solution of Cell Tracker Green CMFDA (5-chloromethylfluorescein diacetate) to a sample of spheroids for 45 minutes at 37°C. After washing, fresh media was applied for 30 minutes before checking growth using a fluorescent microscope.

### 3D invasion studies using breast tumor spheroids or human breast cancer biopsy explants

The collection of fresh breast tumor biopsy material received local ethical approval from the Lothian Tissue Governance Committee (10/S1402/33). Informed consent from patients for the collection and use of tissue samples was obtained prior to use. Breast tumor biopsy material was trimmed and cut into 1mm^3^ pieces. Collagen type I (Wako Chemicals) was mixed with 1:1000 filtered acetic acid, DMEM/F12 (X 10) and 0.22M NaOH in a ratio of 3:5:1:1 (final collagen concentration of 0.3 mg/ml). Compounds in collagen were added to 24-well plate (0.5 ml/well) and 1 piece of tissue added. After incubation at 37°C for 1 hour to solidify the collagen, 500 μl of MEGM complete media (Lonza, Switzerland) containing compound was added per well. Treatments continued for up to 15 days. At termination of experiments, cultures were fixed in 10% phosphate buffered formalin, wax embedded, and processed for immunohistochemistry. 3D spheroid invasion assays were performed in the same manner except that DMEM culture media (prepared as above) was used, and experiments terminated by 48 hours. Results were quantified using Image J Software.

### Immunohistochemistry

Embedded sections from spheroids or tumor explants were dewaxed and antigen retrieval performed before washing and blocking for 10 minutes. After washing, slides were incubated with primary antibody for 1 hour at room temperature or overnight at 4°C, before washing. Slides were incubated with Dako Envision labelled polymer for 30 minutes, washed and treated with DAB substrate, before counterstaining with haematoxylin.

### Tissue microarray (TMA)

Tissue samples originated from patients with primary breast carcinomas treated in the Edinburgh Breast Unit between 1999 and 2002. The study received local ethical approval from the Lothian Research Ethics Committee (08/S1101/41). No informed consent (written or verbal) was obtained for use of retrospective tissue samples from the patients within this study, most of whom were deceased, as this was not deemed necessary by the Ethics Committee. Axillary lymph node dissection was performed on all patients as part of surgery for large or high-grade invasive breast carcinomas and extracted tissues embedded into a recipient paraffin block in a uniformly spaced array pattern for further analysis. TMAs were constructed in biological triplicate. Ninety-two cancers were available for analysis after TMA construction, immunostaining, AQUAsition, and AQUAnalysis as detailed in previous work [48].

### Xenograft studies

The xenograft studies were undertaken under a UK Home Office Project Licence in accordance with the Animals (Scientific Procedures) Act 1986 and studies were approved by the University of Edinburgh Animal Ethics Committee. Adult female nu/nu mice were implanted subcutaneously into both flanks with MDA-MB-231 tumor fragments (previously established from the cell line) and groups of tumors grown to 4–6 mm diameter over a period of approximately 1 month. Animals were allocated to treatment or control groups (10 mice/group) and treatment commenced (defined as day 0). DTP348 (10 mg/kg/day) or FC398A (25mg/kg/day) were given via the intraperitoneal route in saline on days 0–4. Tumor size was measured twice weekly using calipers and the volume calculated according to the formula π/6 × length × width^2^. Relative tumor volumes (%) were calculated for each individual tumor by dividing the tumor volume on day t (Vt) by the tumor volume on day 0 (V0) and multiplying by 100. Tumors were excised and dissected (50% snap frozen in liquid nitrogen; 50% placed into formalin saline for 24 hours) before embedding in paraffin. Ki67 expression was evaluated by immunohistochemistry. Ten fields per xenograft section were evaluated via Image J.

### Statistical analysis

Data was analysed by ANOVA followed by Tukey-Kramer multiple comparison test; a *P* value of < 0.05 was considered statistically significant.

### Other materials

Cell Tracker Green CMFDA (5-Chloromethyl fluorescein Diacetate) was obtained from Invitrogen (UK). HIF-1α antibody was obtained from BD Biosciences (610958); CAIX and NHE1 from Novus Biologicals (NB100-417, and NBP1-20198, respectively); CAIX from Bioscience Slovakia (clone M75; Ki67 from Dako (M7240); cleaved caspase-3 from Cell Signalling (9661); Hypoxyprobe-1 Kit from Hypoxyprobe Inc. All antibodies were validated by western blot to confirm the absence of non-specific bands. The novel ureido-sulfamate CAIX inhibitors FC11409B, FC9398A, FC9403, FC9396A and S4 were synthesized as previously detailed [24, 25]. DTP was synthesized as previously described [26].

## SUPPLEMENTARY FIGURES AND TABLES


