# Supplementary material for: Evaluation of carbonic anhydrase IX as a therapeutic target for inhibition of breast cancer invasion and metastasis using a series of in vitro breast cancer models
Source: Oncotarget. 2015 Jul 3;6(28):24856–70. doi: 10.18632/oncotarget.4498 (PMC4694798; doi:10.18632/oncotarget.4498)
Supplement: Supplementary file 1 [file oncotarget-06-24856-s001.pdf]

## SUPPLEMENTARY FIGURES AND TABLES

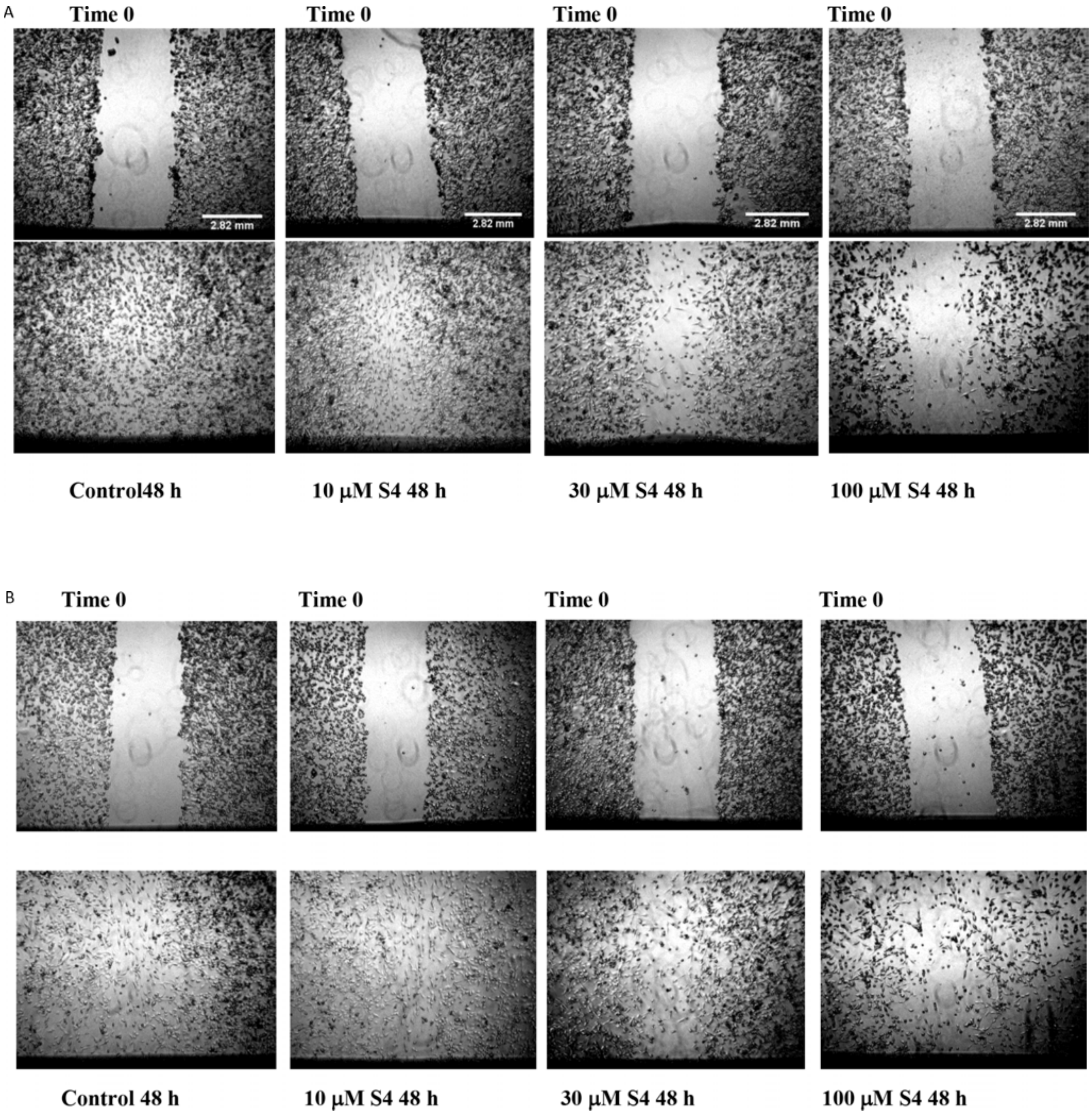


**Supplementary Figure S1: The effect of CAIX inhibitors in 2D wound migrations assays.** Wounding assay using MDA-MB-231 breast cancer cells and S4 in **A.** Normoxia; **B.** Hypoxia. Cells were grown until confluent, and after wounding, treated with S4 at 0 and 48 h timepoints.

*(Continued)*

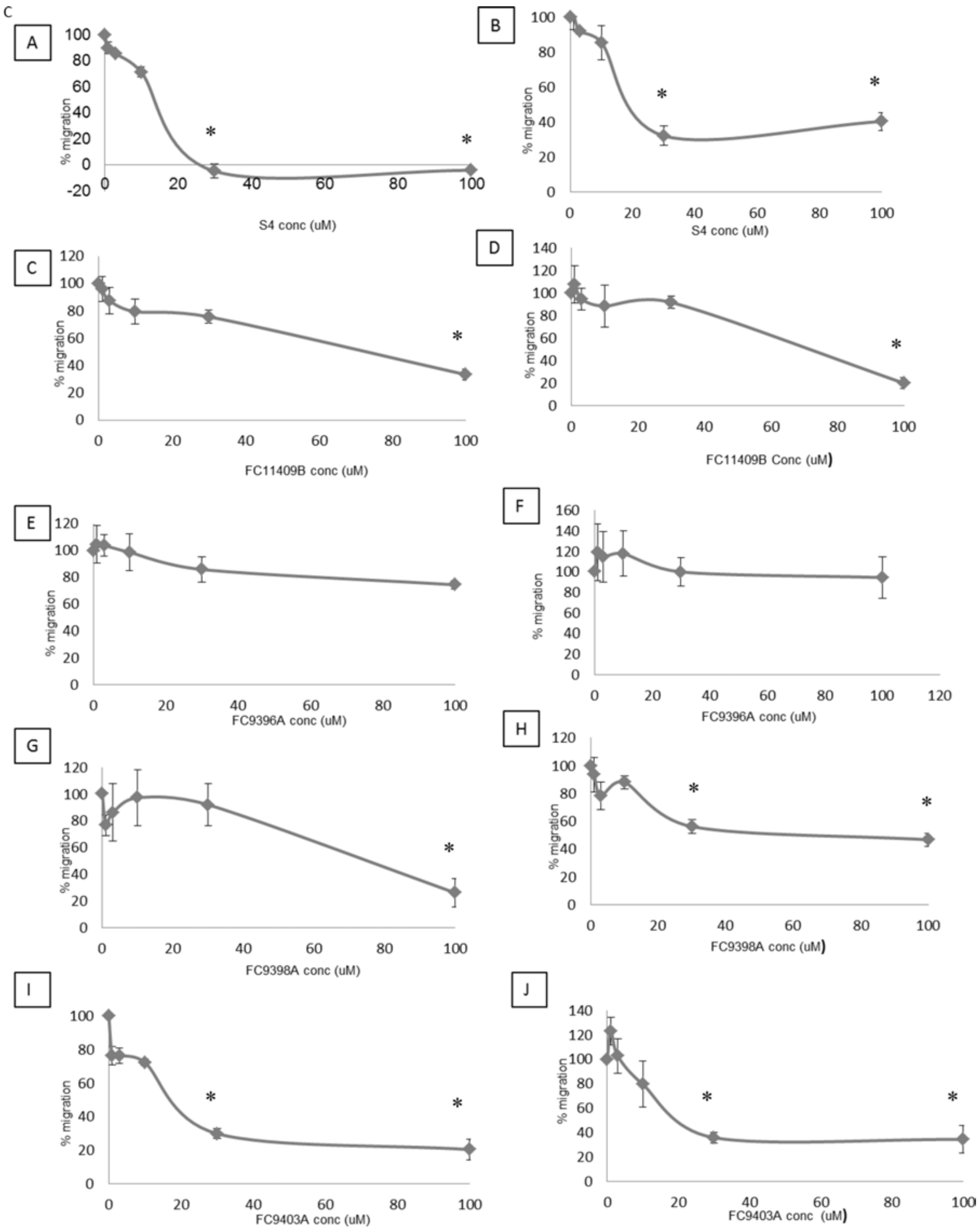


**Supplementary Figure S1: (*Continued*) C.** The effect of SKOV3 migration in wound assays in response to S4, FC11409B in normoxia and hypoxia. A, C, E, G and I, normoxic conditions; B, D, F, H and J hypoxic conditions. All compounds used at 0, 3, 10, 30 and 100 μM ($n$ = 3 for all concentrations). Results shown = mean ± SEM, *$P$ < 0.01.

A

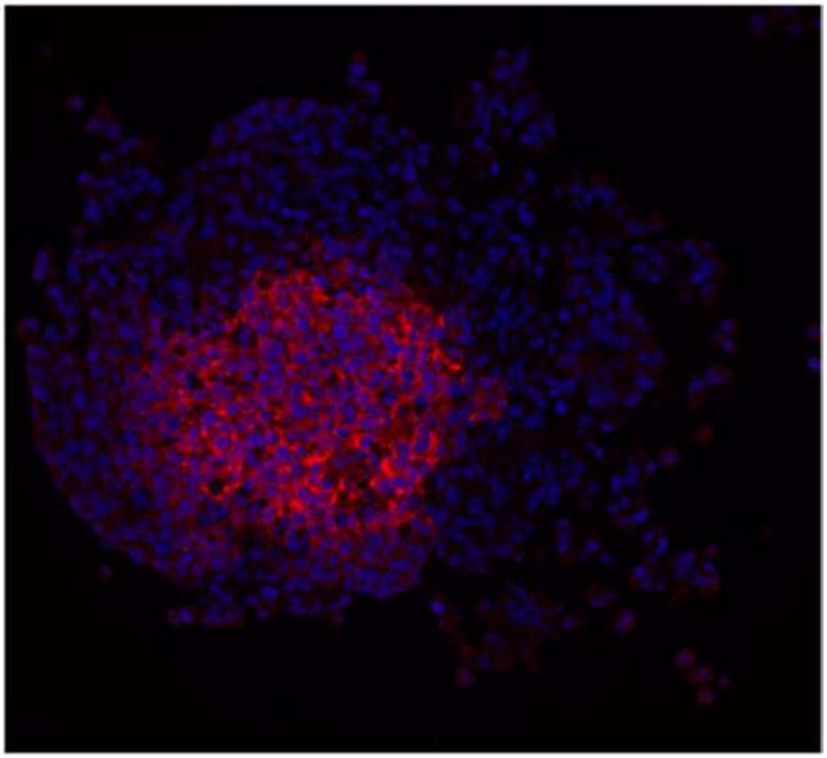

B

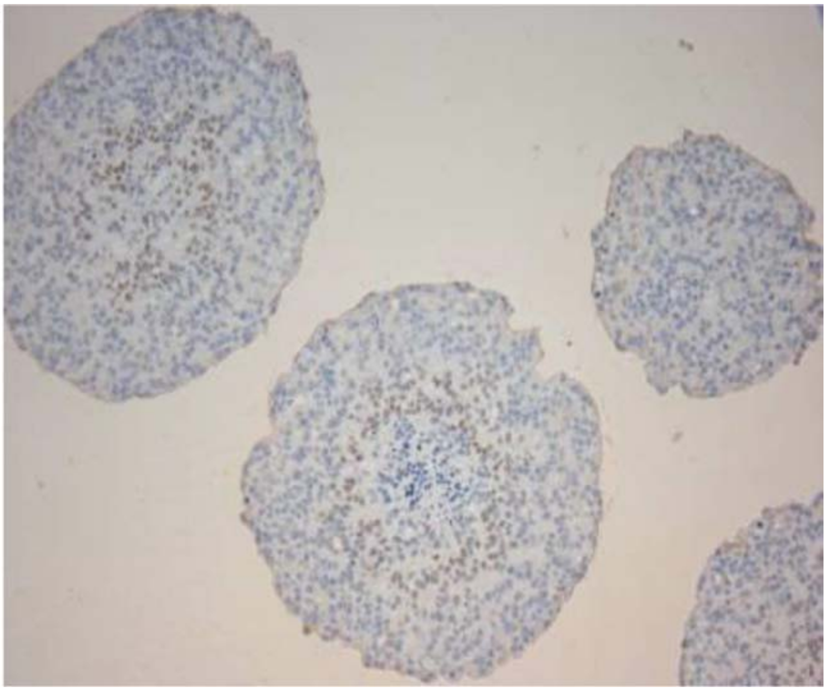

**Supplementary Figure S2: A-C. Immunostaining of 3D breast cancer spheroids.** (A) Hypoxyprobe staining in an MDA-MB-231 breast cancer spheriod; (B) HIF-1α staining in MDA-MB-231 spheroids (1:800);

*(Continued )*

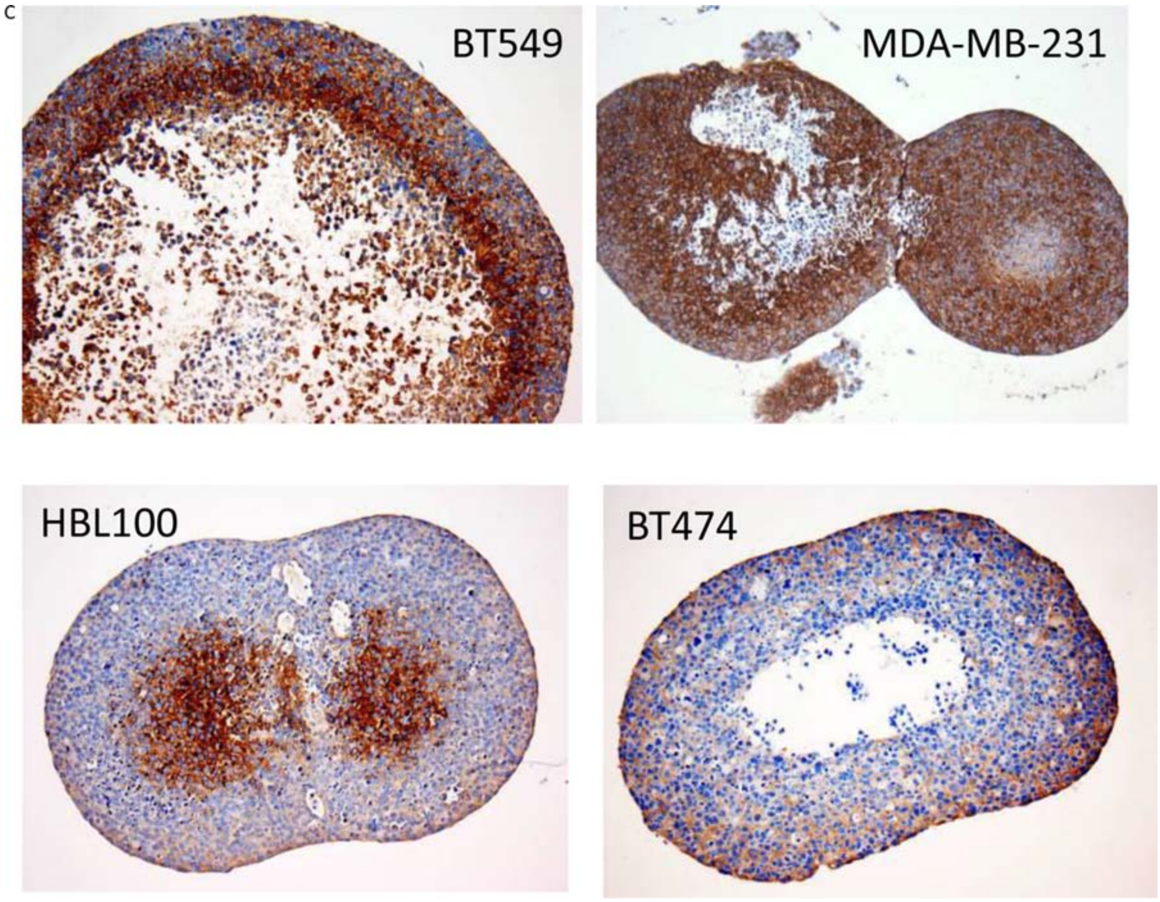


**Supplementary Figure S2: (*Continued*)** (C) CAIX expression in the cell line panel Magnification (x 100).

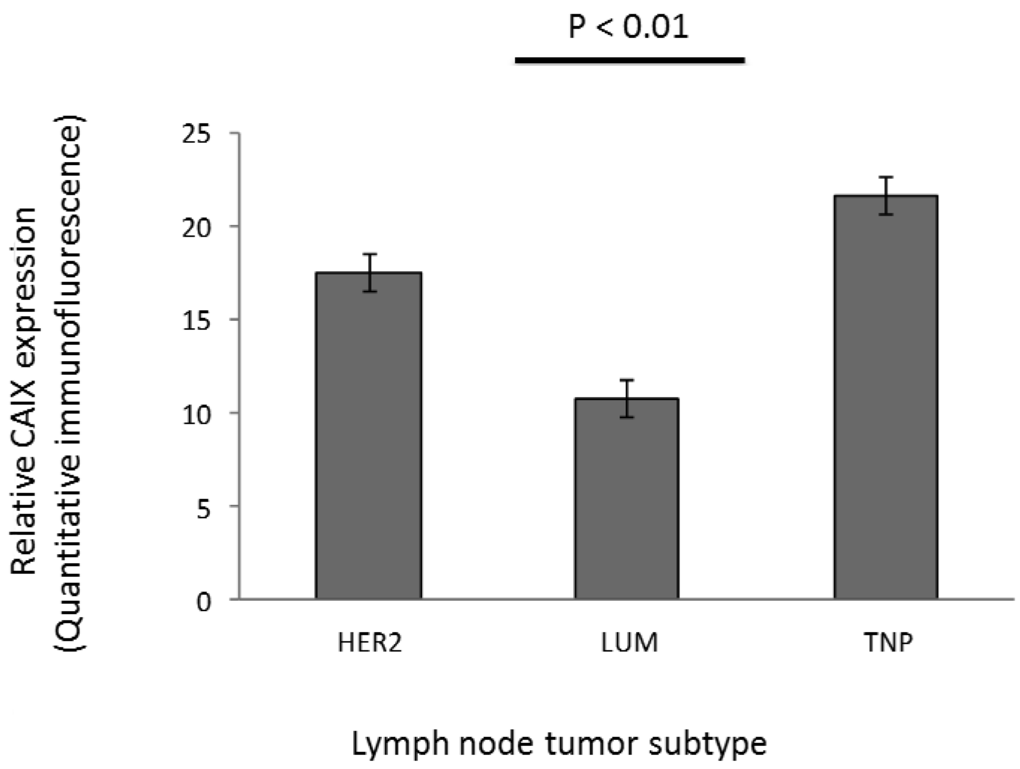


**Supplementary Figure S3: CAIX expression in a series of breast cancer lymph nodes.** AQUAnalysis software was used to calculate target protein expression in HER2 positive (HER2, $n$ = 18); luminal (LUM $n$ = 49); and triple negative breast cancers (TNP, $n$ = 25); *$P$ < 0.01 (ANOVA followed by Tukey test).

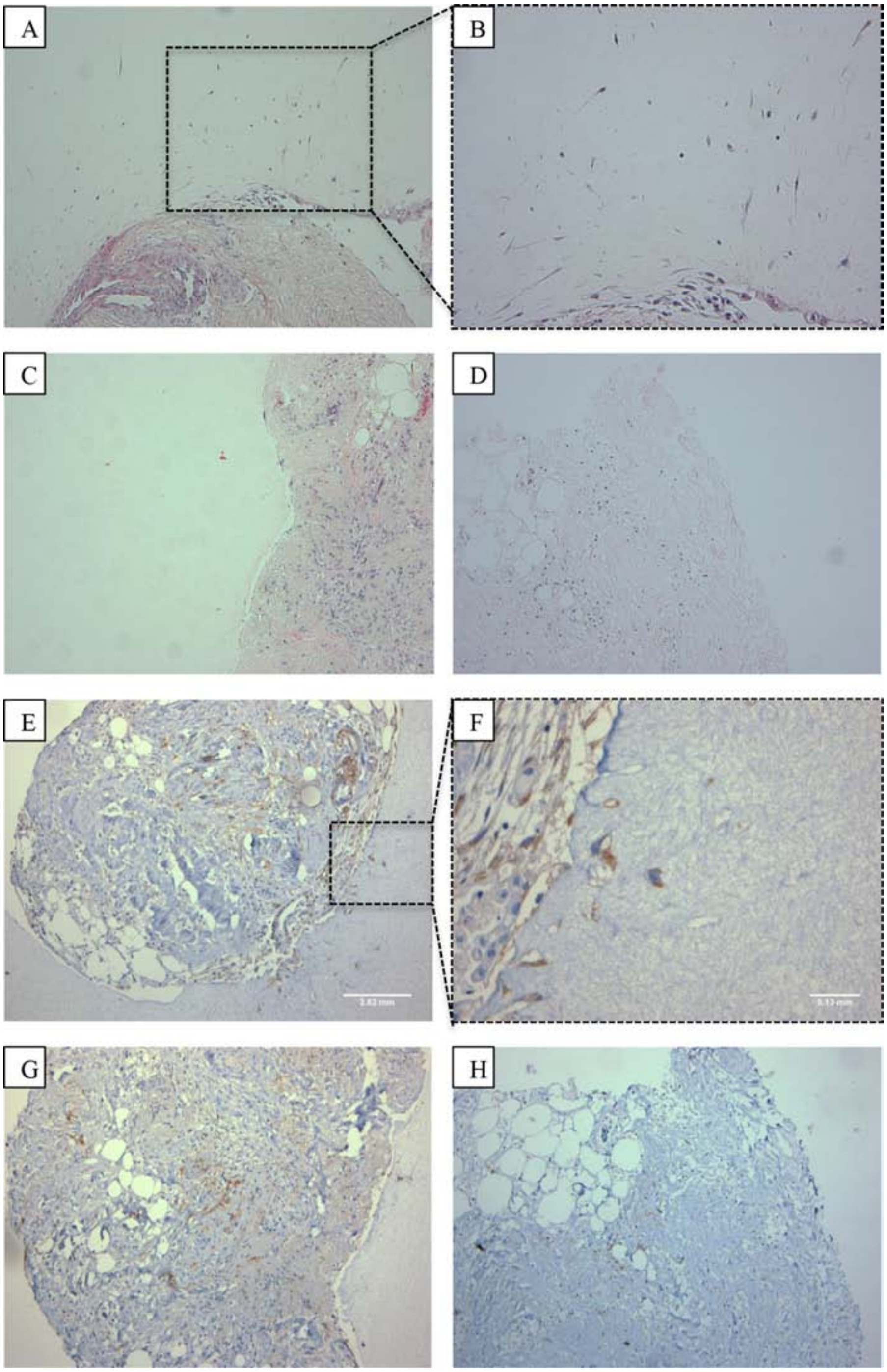


**Supplementary Figure S4: The treatment of breast tumor explants with S4 showing H&E and CAIX staining. A** and **B.** controls; **C.** 30 µM S4; **D.** 100 µM S4; **E** and **F.** invasive control stained for CAIX; **G.** 30 µM S4; **H.** 100 µM S4 stained for CAIX. Explants embedded after 15 days culture. Original magnification of all A, C, D, E and G = x 100; Original magnification of B = x 200; F = x 400; Explants embedded after 15 days culture. Original magnification of A, C and E = x 100; scale bar = 2.82 mm. Original magnification of F = x 400; scale bar = 0.13 mm.

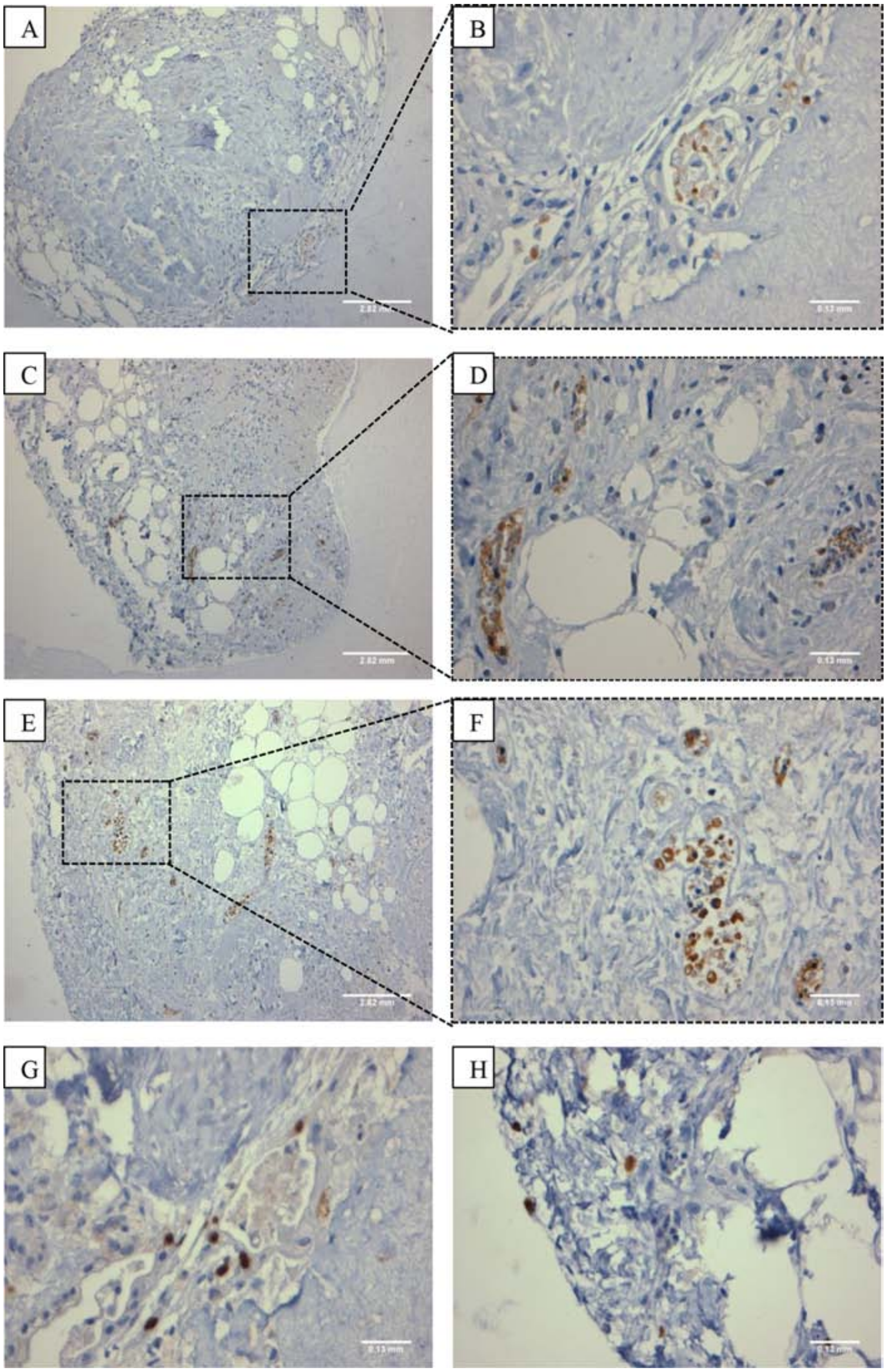

**Supplementary Figure S5: Immunohistochemistry staining for cleaved caspase 3 and Ki67 in treated and untreated explants.** Control explants **A** and **B.** explants treated with 30 μM **C** and **D.** and 100 μM S4 **E** and **F.** all stained for cleaved caspase 3. **G.** 30 μM S4 and **H.** 100 μM S4 treated explants stained for Ki67. Original magnification of A, C and E = x 100; scale bar = 2.82 mm. Original magnification of B, D, and F = x 200; G and H = x 400; scale bar = 0.13 mm.

## Supplementary Table S1: Characteristics of cell line panel

| Cell Line | Receptor Status and | Breast Cancer Subtype |
|---|---|---|
| MCF7 | $ER^+$, $PR^+$ | Luminal |
| MDA.MB.231 | $ER^-$, $PR^-$, $HER2^-$ | Basal B |
| MDA.MB.361 | $ER^+$, $HER2^+$ | Luminal |
| MDA.MB.453 | $ER^-$, $PR^-$ | Luminal |
| MDA.MB.468 | $ER^-$, $PR^-$ | Basal B |
| SKBR3 | $ER^-$, $PR^-$, $HER2^+$ | Luminal |
| T47D | $ER^+$, $PR^+$ | Luminal |
| BT474 | $ER^+$, $PR^+$, $HER2^+$ | Luminal |
| BT549 | $ER^-$, $PR^-$ | Basal B |
| HBL100 | $ER^-$, $PR^-$ | Basal B |
| ZR75/1 | $ER^+$ | Luminal |
| MCF-10A | $ER^-$, $PR^-$ | Basal B |

List of breast cancer cell lines used in this study showing receptor expression and breast cancer subtype adapted from [49].

## Supplementary Table S2: $IC_{50}$ values for the CAIX inhibitors

| Cell line | FC11409B $IC_{50}$ [µM] | FC9398A $IC_{50}$ [µM] | FC9403A $IC_{50}$ [µM] | FC9396A $IC_{50}$ [µM] | S4 $IC_{50}$ [µM] |
|---|---|---|---|---|---|
| MCF7 | 8.4 | 57.8 | 16.1 | 30.6 | 2.02 |
| MDA-MB-231 | 12.9 | 20.2 | 14.8 | 53.5 | 15.6 |
| MDA-MB-361 | 2.6 | 18.2 | 7.4 | 17.1 | 15.1 |
| MDA-MB-453 | 1.7 | 17.4 | 11.6 | 15.1 | 15.8 |
| T47D | 2.8 | 23.2 | 8.8 | 9.0 | 10.1 |
| BT549 | 3.1 | 27.9 | 9.4 | 11.9 | 20.1 |
| HBL100 | 9.1 | 46.4 | 35.7 | 70.8 | 13.9 |

The IC50 values for the compounds on various cell lines in normoxic oxygen concentrations

## Supplementary Table S3: The effect of CAIX inhibitors on migration of MDA-MB-231 cells

| Drug | Normoxia | Hypoxia |
|---|---|---|
| FC11409B | Inhibition at 10 – 100 µM<br>Some cell death at higher concentrations | Inhibition at 10 – 100 µM<br>Some cell death at higher concentrations |
| FC9398A | Inhibition at 10 – 100 µM | Inhibition at 10 – 100 µM |
| FC9403A | Inhibition at 30 – 100 µM | Inhibition at 30 – 100 µM<br>Some cell death at these concentrations |
| FC9396A | Inhibition at 30 – 100 µM | Not effective. |
| S4 | Inhibition at 10 – 100 µM | Inhibition at 10 – 100 µM |

Confluent cell layers were wounded and inhibition of migration by CAIX inhibitors was assessed after 48 h culture in normoxic or hypoxic conditions.
